# Evaluation of the Higher Order Structure of Biotherapeutics Embedded in Hydrogels for Bioprinting and Drug Release

**DOI:** 10.1021/acs.analchem.1c01850

**Published:** 2021-08-02

**Authors:** Domenico Rizzo, Linda Cerofolini, Anna Pérez-Ràfols, Stefano Giuntini, Fabio Baroni, Enrico Ravera, Claudio Luchinat, Marco Fragai

**Affiliations:** † Magnetic Resonance Center (CERM), University of Florence, and Consorzio Interuniversitario Risonanze Magnetiche di Metalloproteine (CIRMMP), Via L. Sacconi 6, Sesto Fiorentino 50019, Italy; ‡ Department of Chemistry “Ugo Schiff”, University of Florence, Via della Lastruccia 3, Sesto Fiorentino 50019, Italy; § Giotto Biotech, S.R.L, Via Madonna del piano 6, Sesto Fiorentino, Florence 50019, Italy; ∥ Analytical Development Biotech Department, Merck Serono S.p.a, Merck KGaA, Guidonia, Rome 00012, Italy

## Abstract

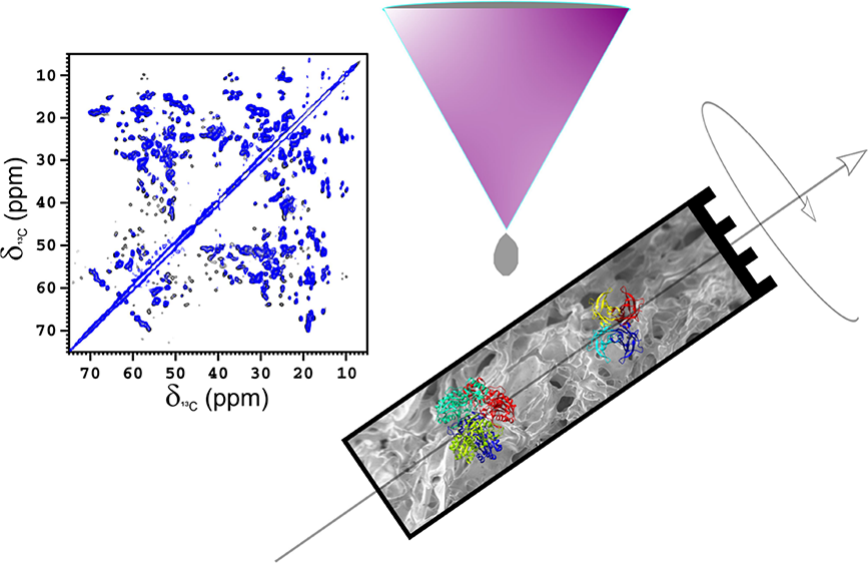

Biocompatible hydrogels for tissue regeneration/replacement and drug release with specific architectures can be obtained by three-dimensional bioprinting techniques. The preservation of the higher order structure of the proteins embedded in the hydrogels as drugs or modulators is critical for their biological activity. Solution nuclear magnetic resonance (NMR) experiments are currently used to investigate the higher order structure of biotherapeutics in comparability, similarity, and stability studies. However, the size of pores in the gel, protein–matrix interactions, and the size of the embedded proteins often prevent the use of this methodology. The recent advancements of solid-state NMR allow for the comparison of the higher order structure of the matrix-embedded and free isotopically enriched proteins, allowing for the evaluation of the functionality of the material in several steps of hydrogel development. Moreover, the structural information at atomic detail on the matrix–protein interactions paves the way for a structure-based design of these biomaterials.

## Introduction

The continuous development of new biocompatible materials is opening new frontiers in medicine and new biotechnological opportunities. Several biomaterials are currently used to replace/support non-functional tissues like those damaged or destroyed by injuries or diseases and in controlled drug release. Materials for tissue regeneration are designed to provide mechanical support to the surrounding tissue, to stimulate cell growth, and to modulate the immune response promoting an extensive cell colonization and matrix reabsorption.^1,2^ Composite scaffolds with a highly resolved architecture, incorporating proteins and seeding cells, can be obtained by three-dimensional (3D) bioprinting techniques starting from biocompatible hydrogels like those formed by hyaluronic acid^3−7^ or mixtures of alginate and gelatine.^6,8−13^ In this context, there is an increasing interest in loading proteins on hydrogels as drugs or modulators of the biological activity.^14−25^ The biological function of a protein is strictly related to its native folding, and the preservation of the higher order structure (HOS) in the composite biomaterial is crucial for its therapeutic function. Actually, the interaction of the protein with the matrix components can alter the protein structure leading to a loss of activity and immunological effects.

Several biophysical methodologies, such as attenuated total reflectance Fourier-transformed infrared and fluorescence spectroscopy, circular dichroism, and differential scanning calorimetry, are usually used to characterize the protein component in heterogeneous materials.^26−28^ However, these analytical methods measure different aspects of the structure, either directly or indirectly, and are often not sensitive enough to small, local changes in the protein fold. Nuclear magnetic resonance (NMR) and mass spectrometry are well-established techniques to investigate the preservation of the HOS of biologics in solution.^29−39^ Solution NMR has been used previously on small proteins and peptides embedded in hydrogels to investigate the folding state in a confined environment^40^ and for the structural characterization through residual dipolar couplings, since hydrogels behave as anisotropic external alignment media.^41−43^ However, when the size of the pores in the gels is too small or strong interactions between the gel matrix and the cargo protein take place, the rotational correlation time of the protein in solution increases and makes solution NMR ineffective in the analysis of the protein structure at the atomic level.

Recently, solid-state NMR has emerged as a tool to characterize the protein component and to reveal protein–matrix interactions in heterogeneous materials. In this respect, the use of solid-state NMR has been described to characterize noncrystalline large protein assemblies,^44−50^ biomaterials,^51,52^ bioinspired silica matrix embedding enzymes,^53−58^ conjugated proteins,^59−62^ protein-grafted nanoparticles,^63^ and vaccines.^64−66^ Here, we prove that solid-state NMR provides detailed information on the preservation of the HOS of proteins embedded into two popular matrices used for 3D bioprinting.

The therapeutic protein *E. coli* asparaginase-II (ANSII), clinically used against acute lymphoblastic leukemia, has recently shown its activity also against solid tumor when administered in long half-life formulations that reduce immunological adverse reactions.^67^

Human transthyretin (TTR) is a physiological protein acting as a hormone carrier.^68,69^ Although some genetic variants of TTR lead to a systemic amyloidosis called familial amyloid polyneuropathy,^70^ TTR is a potential drug carrier and has been recently proposed as a multivalency Fab platform for target clustering.^71^

Therefore, these two proteins are suitable models to investigate how the matrices used for 3D bioprinting interplay with embedded proteins and are used here to prove the potential of solid-state NMR (SSNMR) in the characterization of the protein components during the design of these composite hydrogels.

## Experimental Section

### Sample Preparation and NMR Measurements

[U-^13^C-^15^N] ANSII was expressed and purified as previously described.^59,61−64^ The expression and purification protocol of [U-^13^C-^15^N] TTR is reported in the Supporting Information. All the hydrogels embedding the selected proteins (ANSII and TTR) were directly generated in Bruker 3.2 mm thin-walls zirconia rotors with bottom and top caps, starting from the dried materials prepared by using the different procedures described below.

The sample of [U-^13^C-^15^N] ANSII encapsulated in the alginate/gelatine hydrogel was prepared by incorporating the freeze-dried protein (4 mg) into a mixture of 1:1 alginate/gelatine powders (5 mg) and then by rehydrating the dried mixture within the rotor.^72^ A different procedure was used to prepare the sample of [U-^13^C-^15^N] TTR encapsulated in the alginate/gelatine hydrogel. The dry mixture containing TTR was prepared by lyophilizing a solution containing all the components (6 mg of protein and 5 mg of the 1:1 alginate/gelatine mixture). In both cases, the dried material was packed in the rotor and hydrated with MilliQ H_2_O to reach a final concentration of ∼5–7% w/w for alginate and gelatine. Finally, a concentrated solution of CaCl_2_ (to reach a concentration of 100 mM in the rotor) was added to cross-link the hydrogel materials within the rotor.^73,74^

A sample of [U-^13^C-^15^N] TTR protein encapsulated in the alginate/gelatine hydrogel was also analyzed by solution NMR. The gel was prepared by dissolving a mixture of alginate and gelatine (∼7% w/w) in 600 μL of a solution of TTR (100 μM in 50 mM MES, pH 6.5, 100 mM NaCl, 5 mM DTT). Then, the material was transferred in a 5 mm tube and cross-linked by adding a concentrated solution of CaCl_2_ (to reach a concentration of 100 mM) in the NMR tube. The 2D ^1^H-^15^N TROSY-HSQC spectrum recorded on the encapsulated protein was superimposed with that of TTR collected in solution (see Figure S1).

The hyaluronic acid hydrogels encapsulating the selected proteins ([U-^13^C-^15^N] ANSII or TTR) were prepared by packing the rotor with consecutive layers of the freeze-dried protein (∼4–6 mg) and freeze-dried hyaluronic acid (Jonexa, 7–9 mg), which had been previously dialyzed against MilliQ H_2_O to remove the excess of salts. The material was finally rehydrated with MilliQ H_2_O (from 10 to 20 μL). Sample homogeneity was obtained after rotor spinning and supported by the quality of the spectra that suggests the presence of a protein experiencing a single environment.

Samples of freeze-dried proteins were prepared as reference. The free proteins (∼20 and 25 mg of ANSII and TTR, respectively) were freeze-dried in the presence of PEG1000 (4 and 2.5 mg for ANSII and TTR, respectively); the materials were packed into a Bruker 3.2 mm zirconia rotor and rehydrated with MilliQ H_2_O (∼9 and 16 μL for ANSII and TTR, respectively). CaCl_2_ was not present in the samples of rehydrated freeze-dried proteins.

Silicon plugs (courtesy of Bruker Biospin) placed below the turbine cap were used to close the rotor and preserve hydration.

SSNMR experiments were recorded on a Bruker Avance III spectrometer operating at 800 MHz (18.8 T, 201.2 MHz ^13^C Larmor frequency) equipped with a Bruker 3.2 mm Efree NCH probe-head. The spectra were recorded at 14 kHz MAS frequency, and the sample temperature was kept at ∼290 K. The sample of the alginate/gelatine hydrogel encapsulating TTR was also investigated at a higher spinning frequency (16 and 20 kHz).

Standard ^13^C-detected SSNMR spectra (2D ^15^N-^13^C NCA, ^15^N-^13^C NCO, and ^13^C-^13^C DARR, mixing time 50 ms) were acquired on all the samples (except for TTR encapsulated in the alginate/gelatine hydrogel) using the pulse sequences reported in the literature.^75^ 2D ^13^C-^13^C CORDxy4^76^ was instead recorded for the sample of the alginate/gelatine hydrogel encapsulating TTR at a higher frequency speed (20 kHz), to favor the protein sedimentation.

All the spectra were processed with the Bruker TopSpin 3.2 software package and analyzed with the program CARA.^77^

## Results and Discussion

### Analysis of the Preservation of the HOS of the Proteins Encapsulated in the Hyaluronic Acid Hydrogel by SSNMR

The selected proteins (ANSII and TTR) encapsulated in the hyaluronic acid hydrogel (ANSII-HA and TTR-HA, respectively) were first analyzed by SSNMR. The 1D {^1^H}-^13^C cross polarization spectra of ANSII-HA and TTR-HA show well-resolved and sharp signals with quality comparable with that of the spectra of the rehydrated freeze-dried materials (Figure S2).

**Figure 1:**
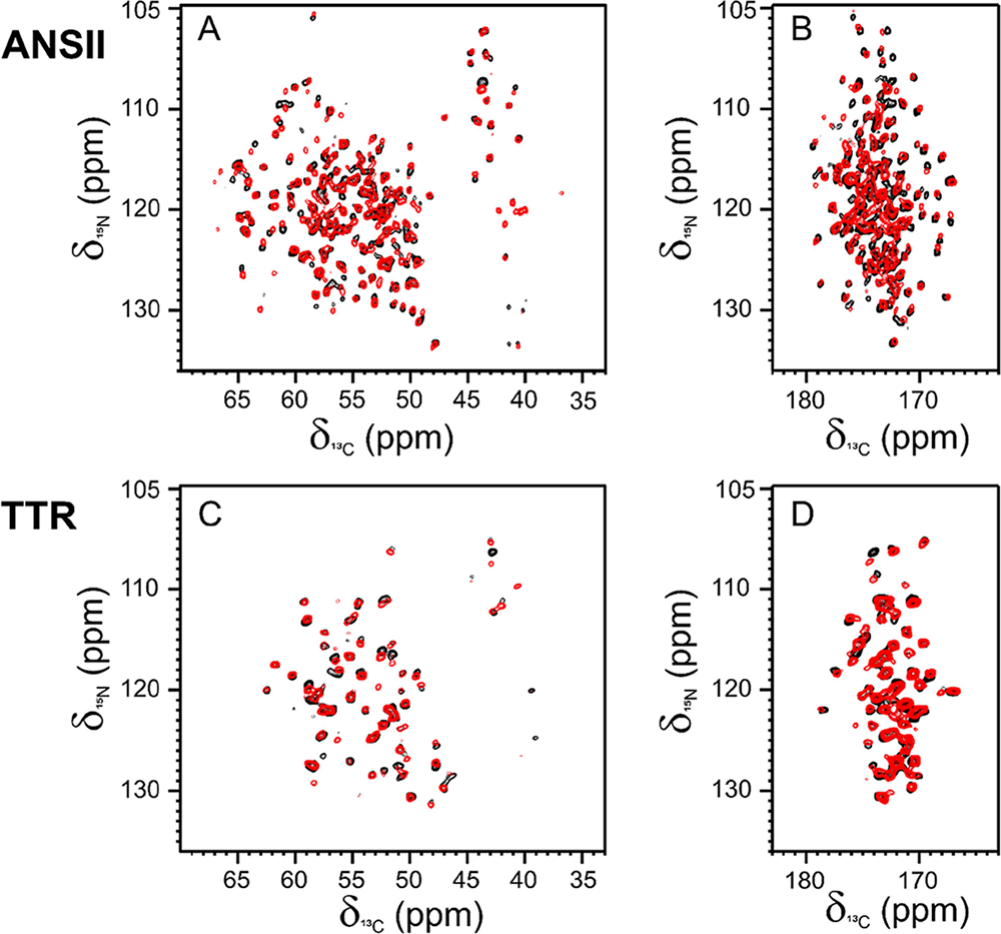
(A, C) 2D ^15^N ^13^C NCA and (B, D) NCO spectra of ANSII-HA (red, top) and TTR-HA (red, bottom) superimposed with NCA and NCO of the rehydrated freeze-dried reference proteins (black). The spectra were acquired at ∼290 K, MAS 14 kHz and 800 MHz.

Despite the limited concentration of the embedded proteins in the hydrogel, the 2D amide-carbon alpha (2D ^15^N ^13^C NCA) and amide-carbonyl (2D ^15^N ^13^C NCO) correlation spectra of ANSII-HA (Figure 1A,B) and TTR-HA (Figure 1C,D) are of high quality and comparable, for the number of cross-peaks detected, with those of rehydrated freeze-dried proteins. For both proteins embedded in the hyaluronic acid matrix, the matching of the resonances of the 2D-NMR spectral fingerprints with those of their own reference allows us to assess the preservation of the HOS after encapsulation in the matrix.

**Figure 2:**
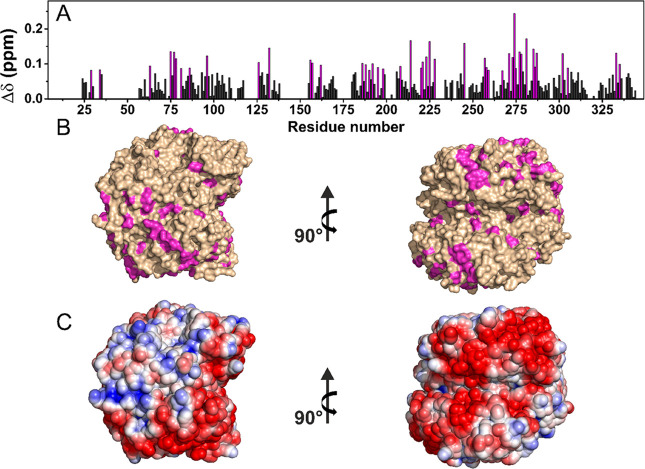
(A) Chemical shift perturbations (CSPs) of ANSII-HA with respect to rehydrated freeze-dried ANSII, evaluated according to the formula . The residues experiencing the largest variations have been highlighted in magenta. (B) CSP mapping on the protein surface (PDB code: 3ECA) with the region with the largest perturbation in magenta. (C) Electrostatic potential generated by APBS plugin in PyMOL on 3ECA with blue and red representing the regions of positive and negative electrostatic potential, respectively.

**Figure 3:**
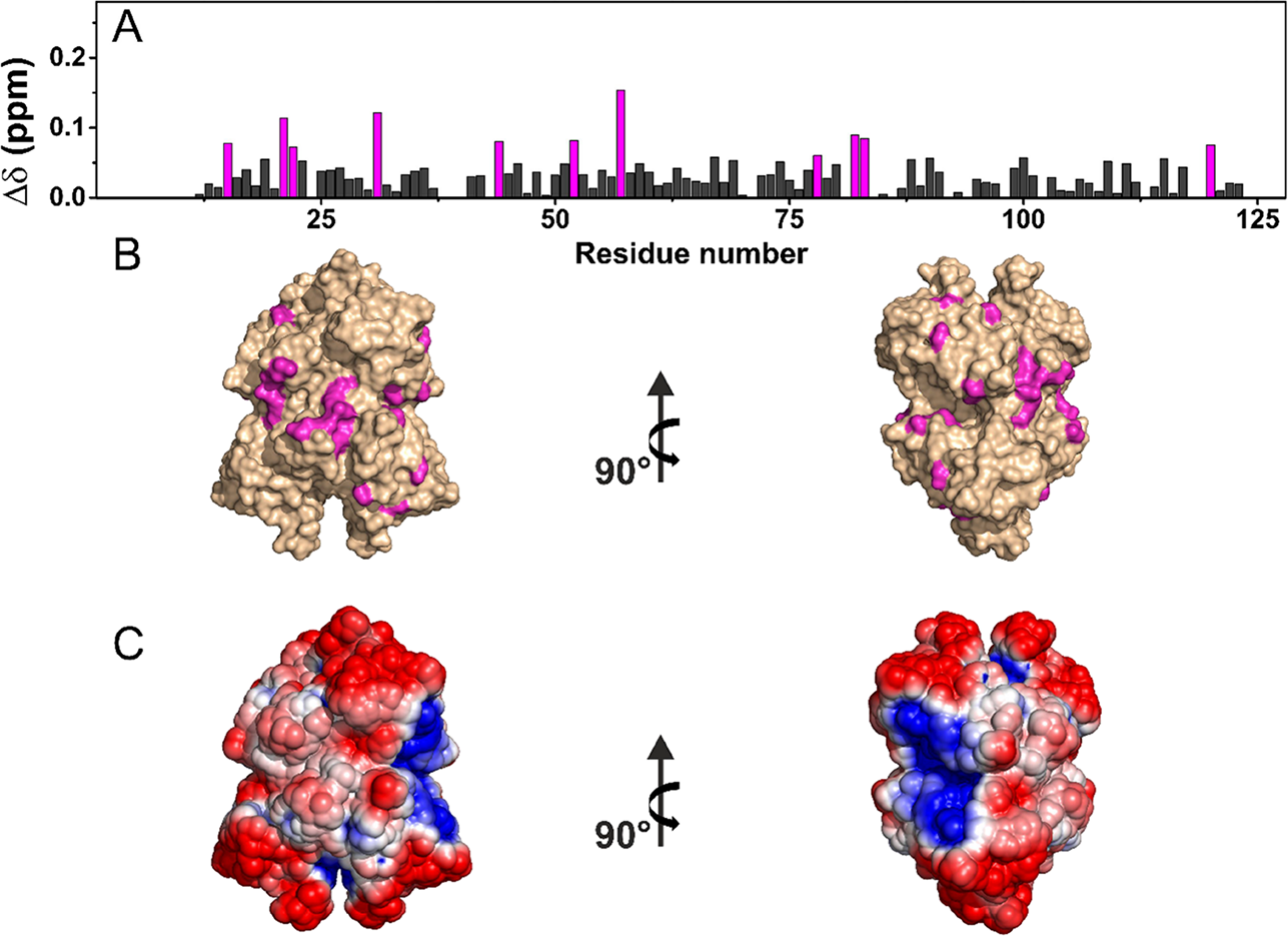
(A) Chemical shift perturbations (CSPs) of TTR-HA with respect to rehydrated freeze-dried TTR, evaluated according to the formula . The residues experiencing the largest variations have been highlighted in magenta. (B) CSP mapping on the protein surface (PDB code: 1BMZ) with the region with the largest perturbation in magenta. (C) Electrostatic potential generated by APBS plugin in PyMOL on 1BMZ with blue and red representing the regions of positive and negative electrostatic potential, respectively.

The assignment of the 2D ^15^N ^13^C NCA and NCO spectra of ANSII-HA and TTR-HA was easily obtained by comparison with the 2D ^15^N ^13^C NCA and NCO collected for the rehydrated freeze-dried proteins and also using the information from the 2D ^13^C-^13^C correlation spectrum (dipolar assisted rotational resonance, DARR) acquired for ANSII-HA and TTR-HA. The analyses of the chemical shift perturbation (CSP) of the NCA spectra of the proteins embedded in the hyaluronic acid hydrogels, with respect to the NCA of the corresponding rehydrated freeze-dried references, are reported in Figures 2 and 3. Most CSP values were less than 0.1 ppm for ANSII-HA and even lower for TTR-HA. The analysis of the CSPs shows that for ANSII-HA, hydrophobic (Ala, Val, Ile, Tyr, and Phe) and neutral polar (Thr, Ser, Asn, and Gln) residues experience the largest effects (Figure 2). Minimal CSPs were observed in TTR-HA protein where the largest effects again involve hydrophobic residues and neutral polar surface patches (Figure 3).

### Analysis of the Preservation of the HOS of the Proteins Encapsulated in the Alginate/Gelatine Hydrogel by SSNMR

The same analysis was also performed on the alginate/gelatine hydrogels encapsulating ANSII and TTR, respectively (ANSII-AG and TTR-AG). The 1D {^1^H}-^13^C cross polarization spectra of ANSII-AG and TTR-AG show the same spreading of the resonances of the corresponding rehydrated freeze-dried analogue. However, in particular for TTR-AG, the signals feature broader lines than in the rehydrated freeze-dried protein (Figure S2).

**Figure 4:**
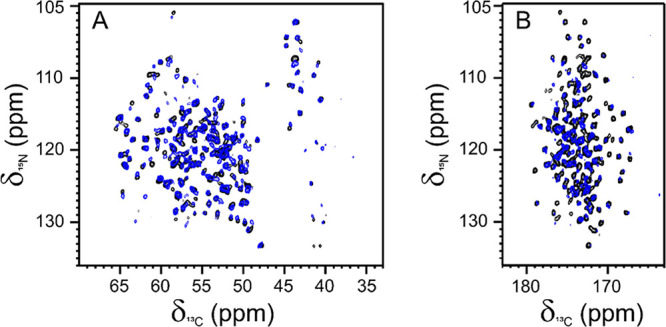
(A) 2D ^15^N ^13^C NCA and (B) NCO spectra of ANSII-AG (blue) superimposed with the NCA and NCO of the rehydrated freeze-dried reference protein (black). The spectra were acquired at ∼290 K, MAS 14 kHz and 800 MHz.

The NCA and NCO correlation spectra collected for ANSII-AG (Figure 4) are still of high quality and comparable, for the number of cross-peaks detected, with those collected on rehydrated freeze-dried ANSII. On the contrary, for TTR-AG, the fast decay of the NMR signal does not allow us to collect high quality and well-resolved 2D spectra. However, by increasing the spinning rate up to 16 and 20 kHz, the signals become sharper and increase in intensity (Figure S3), indicating a more efficient protein immobilization. Therefore, it was possible to acquire a 2D ^13^C-^13^C correlation spectrum at 20 kHz, which allowed us to assess the folding state of the protein in the hydrogel and, after comparison with that acquired for the rehydrated freeze-dried reference (Figure S4), confirm the preservation of the HOS after encapsulation. The structural analysis of TTR encapsulated in the alginate/gelatine matrix was also attempted using solution NMR. However, all the signals, but the N- and C-termini (Thr3-Ser8; Lys126-Glu127), are broadened beyond detection (Figure S1).

**Figure 5:**
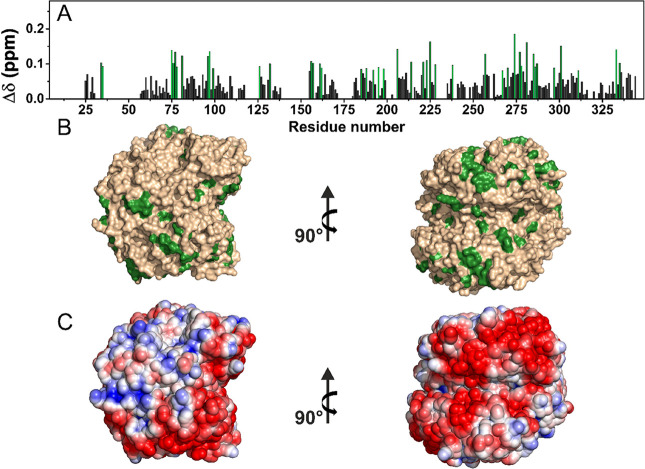
(A) Chemical shift perturbations (CSPs) of ANSII-AG with respect to rehydrated freeze-dried ANSII, evaluated according to the formula . The residues experiencing the largest variations have been highlighted in green. (B) CSP mapping on the protein surface (PDB code: 3ECA) with the region with the largest perturbation in green. (C) Electrostatic potential generated by APBS plugin in PyMOL on 3ECA with blue and red representing the regions of positive and negative electrostatic potential, respectively.

The assignment of the ANSII-AG spectra could be easily obtained by comparison with the spectra collected for the rehydrated freeze-dried protein and complemented with the information from the 2D ^13^C-^13^C correlation spectrum acquired for ANSII-AG. The analysis of the CSP of the NCA spectrum of ANSII-AG with respect to the NCA of the rehydrated freeze-dried reference is reported in Figure 5.

The analysis of the CSPs shows that also for ANSII-AG, hydrophobic (Ala, Val, Ile, Tyr, and Phe) and neutral polar (Thr, Ser, Ans, and Gln) residues experience the largest effects. In particular, many threonine residues are affected by significant CSP, thus suggesting a possible interaction of these surface residues with the hydroxyl groups of alginate in the hydrogel.

Collectively, the good superimposition of the spectra and the small CSPs observed for the two proteins prove the preservation of their native HOS, thus providing the first fundamental information on the investigated biomaterial. Additional information on protein–matrix interactions is obtained from the line broadening of the signals in the spectra. For TTR, the large line broadening, its dependence from the spinning rate, and the small CSPs suggest a weaker protein–matrix interaction with respect to ANSII protein, although the different molecular weights may also play a role. The different behavior is probably related to the different sizes of the proteins and to the physical–chemical properties of the surface due to the different amino acid compositions. In this respect, the observation that hydrophobic and polar neutral amino acids on the protein surface experience the largest effects provides a way to design possible chemical modifications of the matrix in order to tune the protein–matrix interactions and the properties of the resulting biomaterial.^78−82^

## Conclusions

In summary, we demonstrate that 2D-SSNMR spectra can be exploited to assess the preservation of HOS of proteins when embedded in matrices used for 3D bioprinting and drug release. This analytical method can be integrated in the pipeline for the development of new composite hydrogels bearing biotherapeutics. In particular, when the assignment is available, the analysis of the residues experiencing chemical shift variations can provide information for a quality by design approach of these innovative biomaterials.

## Supporting Information Available

Protocols for the expression and purification of isotopically enriched *E. coli* asparaginase-II (ANSII) and human transthyretin (TTR) and figures showing additional spectra of *E. coli* asparaginase-II (ANSII) and human transthyretin (TTR) (PDF)

## References

[ref1] Gaharwar A. K.; Singh I.; Khademhosseini A. Engineered Biomaterials for in Situ Tissue Regeneration. Nat. Rev. Mater. 2020, 5, 686–705. 10.1038/s41578-020-0209-x.

[ref2] Gu L.; Mooney D. J. Biomaterials and Emerging Anticancer Therapeutics: Engineering the Microenvironment. Nat. Rev. Cancer 2016, 16, 56–66. 10.1038/nrc.2015.3. 26694936 PMC4790726

[ref3] Suri S.; Han L.-H.; Zhang W.; Singh A.; Chen S.; Schmidt C. E. Solid Freeform Fabrication of Designer Scaffolds of Hyaluronic Acid for Nerve Tissue Engineering. Biomed. Microdevices 2011, 13, 983–993. 10.1007/s10544-011-9568-9. 21773726 PMC8638827

[ref4] Noh I.; Kim N.; Tran H. N.; Lee J.; Lee C. 3D Printable Hyaluronic Acid-Based Hydrogel for Its Potential Application as a Bioink in Tissue Engineering. Biomater. Res. 2019, 23, 3 10.1186/s40824-018-0152-8. 30774971 PMC6364434

[ref5] Wei Y.-T.; He Y.; Xu C.-L.; Wang Y.; Liu B.-F.; Wang X.-M.; Sun X.-D.; Cui F.-Z.; Xu Q.-Y. Hyaluronic Acid Hydrogel Modified with Nogo-66 Receptor Antibody and Poly-L-Lysine to Promote Axon Regrowth after Spinal Cord Injury. J. Biomed. Mater. Res. B Appl. Biomater. 2010, 95B, 110–117. 10.1002/jbm.b.31689. 20725955

[ref6] Antich C.; de Vicente J.; Jiménez G.; Chocarro C.; Carrillo E.; Montañez E.; Gálvez-Martín P.; Marchal J. A. Bio-Inspired Hydrogel Composed of Hyaluronic Acid and Alginate as a Potential Bioink for 3D Bioprinting of Articular Cartilage Engineering Constructs. Acta Biomater. 2020, 106, 114–123. 10.1016/j.actbio.2020.01.046. 32027992

[ref7] Skardal A.; Zhang J.; Prestwich G. D. Bioprinting Vessel-like Constructs Using Hyaluronan Hydrogels Crosslinked with Tetrahedral Polyethylene Glycol Tetracrylates. Biomaterials 2010, 31, 6173–6181. 10.1016/j.biomaterials.2010.04.045. 20546891

[ref8] Chung J. H. Y.; Naficy S.; Yue Z.; Kapsa R.; Quigley A.; Moulton S. E.; Wallace G. G. Bio-Ink Properties and Printability for Extrusion Printing Living Cells. Biomater. Sci. 2013, 1, 763–773. 10.1039/c3bm00012e. 32481829

[ref9] Wu Z.; Su X.; Xu Y.; Kong B.; Sun W.; Mi S. Bioprinting Three-Dimensional Cell-Laden Tissue Constructs with Controllable Degradation. Sci. Rep. 2016, 6, 24474 10.1038/srep24474. 27091175 PMC4835808

[ref10] Poldervaart M. T.; Wang H.; van der Stok J.; Weinans H.; Leeuwenburgh S. C. G.; Öner F. C.; Dhert W. J. A.; Alblas J. Sustained Release of BMP-2 in Bioprinted Alginate for Osteogenicity in Mice and Rats. PLoS One 2013, 8, e72610 10.1371/journal.pone.0072610. 23977328 PMC3747086

[ref11] Duan B.; Hockaday L. A.; Kang K. H.; Butcher J. T. 3D Bioprinting of Heterogeneous Aortic Valve Conduits with Alginate/Gelatin Hydrogels. J. Biomed. Mater. Res. A 2013, 101A, 1255–1264. 10.1002/jbm.a.34420. PMC3694360 23015540

[ref12] Giuseppe M. D.; Law N.; Webb B.; Macrae R. A.; Liew L. J.; Sercombe T. B.; Dilley R. J.; Doyle B. J. Mechanical Behaviour of Alginate-Gelatin Hydrogels for 3D Bioprinting. J. Mech. Behav. Biomed. Mater. 2018, 79, 150–157. 10.1016/j.jmbbm.2017.12.018. 29304429

[ref13] Jia W.; Gungor-Ozkerim P. S.; Zhang Y. S.; Yue K.; Zhu K.; Liu W.; Pi Q.; Byambaa B.; Dokmeci M. R.; Shin S. R.; Khademhosseini A. Direct 3D Bioprinting of Perfusable Vascular Constructs Using a Blend Bioink. Biomaterials 2016, 106, 58–68. 10.1016/j.biomaterials.2016.07.038. 27552316 PMC5300870

[ref14] Buwalda S. J.; Vermonden T.; Hennink W. E. Hydrogels for Therapeutic Delivery: Current Developments and Future Directions. Biomacromolecules 2017, 18, 316–330. 10.1021/acs.biomac.6b01604. 28027640

[ref15] Chen J.; Ouyang J.; Chen Q.; Deng C.; Meng F.; Zhang J.; Cheng R.; Lan Q.; Zhong Z. EGFR and CD44 Dual-Targeted Multifunctional Hyaluronic Acid Nanogels Boost Protein Delivery to Ovarian and Breast Cancers In Vitro and In Vivo. ACS Appl. Mater. Interfaces 2017, 9, 24140–24147. 10.1021/acsami.7b06879. 28675028

[ref16] Zhu Q.; Chen X.; Xu X.; Zhang Y.; Zhang C.; Mo R. Tumor-Specific Self-Degradable Nanogels as Potential Carriers for Systemic Delivery of Anticancer Proteins. Adv. Funct. Mater. 2018, 28, 1707371 10.1002/adfm.201707371.

[ref17] Wawrzyńska E.; Kubies D. Alginate Matrices for Protein Delivery – a Short Review. Physiol. Res. 2018, S319–S334. 10.33549/physiolres.933980. 30379553

[ref18] Iqbal S.; Blenner M.; Alexander-Bryant A.; Larsen J. Polymersomes for Therapeutic Delivery of Protein and Nucleic Acid Macromolecules: From Design to Therapeutic Applications. Biomacromolecules 2020, 21, 1327–1350. 10.1021/acs.biomac.9b01754. 32078290

[ref19] Shigemitsu H.; Kubota R.; Nakamura K.; Matsuzaki T.; Minami S.; Aoyama T.; Urayama K.; Hamachi I. Protein-Responsive Protein Release of Supramolecular/Polymer Hydrogel Composite Integrating Enzyme Activation Systems. Nat. Commun. 2020, 11, 3859 10.1038/s41467-020-17698-0. 32737298 PMC7395795

[ref20] Lau C. M. L.; Jahanmir G.; Yu Y.; Chau Y. Controllable Multi-Phase Protein Release from in-Situ Hydrolyzable Hydrogel. J. Controlled Release 2021, 335, 75–85. 10.1016/j.jconrel.2021.05.006. 33971140

[ref21] Tae H.; Lee S.; Ki C. S. β-Glucan Hybridized Poly (Ethylene Glycol) Microgels for Macrophage-Targeted Protein Delivery. J. Ind. Eng. Chem. 2019, 75, 69–76. 10.1016/j.jiec.2019.02.014.

[ref22] Chang D.; Park K.; Famili A. Hydrogels for Sustained Delivery of Biologics to the Back of the Eye. Drug Discovery Today 2019, 24, 1470–1482. 10.1016/j.drudis.2019.05.037. 31202673

[ref23] Ziegler C. E.; Graf M.; Beck S.; Goepferich A. M. A Novel Anhydrous Preparation of PEG Hydrogels Enables High Drug Loading with Biologics for Controlled Release Applications. Eur. Polym. J. 2021, 147, 110286 10.1016/j.eurpolymj.2021.110286.

[ref24] Gombotz W. R.; Wee S. F. Protein Release from Alginate Matrices. Adv. Drug Delivery Rev. 2012, 64, 194–205. 10.1016/j.addr.2012.09.007. 10837629

[ref25] Vermonden T.; Censi R.; Hennink W. E. Hydrogels for Protein Delivery. Chem. Rev. 2012, 112, 2853–2888. 10.1021/cr200157d. 22360637

[ref26] Kumar S.; Koh J. Physiochemical, Optical and Biological Activity of Chitosan-Chromone Derivative for Biomedical Applications. Int. J. Mol. Sci. 2012, 13, 6102–6116. 10.3390/ijms13056102. 22754352 PMC3382751

[ref27] Dong A.; Jones L. S.; Kerwin B. A.; Krishnan S.; Carpenter J. F. Secondary Structures of Proteins Adsorbed onto Aluminum Hydroxide: Infrared Spectroscopic Analysis of Proteins from Low Solution Concentrations. Anal. Biochem. 2006, 351, 282–289. 10.1016/j.ab.2006.01.008. 16460655

[ref28] Kirkitadze M.; Sinha A.; Hu J.; Williams W.; Cates G. Adjuvanted Vaccine Components: Analysis of Structure and Stability. Procedia Vaccinol. 2009, 1, 135–139. 10.1016/j.provac.2009.07.025.

[ref29] Arbogast L. W.; Brinson R. G.; Marino J. P. Mapping Monoclonal Antibody Structure by 2D 13C NMR at Natural Abundance. Anal. Chem. 2015, 87, 3556–3561. 10.1021/ac504804m. 25728213

[ref30] Brinson R. G.; Marino J. P.; Delaglio F.; Arbogast L. W.; Evans R. M.; Kearsley A.; Gingras G.; Ghasriani H.; Aubin Y.; Pierens G. K.; Jia X.; Mobli M.; Grant H. G.; Keizer D. W.; Schweimer K.; Ståhle J.; Widmalm G.; Zartler E. R.; Lawrence C. W.; Reardon P. N.; Cort J. R.; Xu P.; Ni F.; Yanaka S.; Kato K.; Parnham S. R.; Tsao D.; Blomgren A.; Rundlöf T.; Trieloff N.; Schmieder P.; Ross A.; Skidmore K.; Chen K.; Keire D.; Freedberg D. I.; Suter-Stahel T.; Wider G.; Ilc G.; Plavec J.; Bradley S. A.; Baldisseri D. M.; Sforça M. L.; de Zeri A. C. M.; Wei J. Y.; Szabo C. M.; Amezcua C. A.; Jordan J. B.; Wikström M. Enabling Adoption of 2D-NMR for the Higher Order Structure Assessment of Monoclonal Antibody Therapeutics. mAbs 2019, 11, 94–105. 10.1080/19420862.2018.1544454. 30570405 PMC6343768

[ref31] Jones L. M.; Zhang H.; Cui W.; Kumar S.; Sperry J. B.; Carroll J. A.; Gross M. L. Complementary MS Methods Assist Conformational Characterization of Antibodies with Altered S-S Bonding Networks. J. Am. Soc. Mass Spectrom. 2013, 24, 835–845. 10.1007/s13361-013-0582-4. 23483515 PMC3651811

[ref32] Pan L. Y.; Salas-Solano O.; Valliere-Douglass J. F. Conformation and Dynamics of Interchain Cysteine-Linked Antibody-Drug Conjugates as Revealed by Hydrogen/Deuterium Exchange Mass Spectrometry. Anal. Chem. 2014, 86, 2657–2664. 10.1021/ac404003q. 24512515

[ref33] Ehkirch A.; Hernandez-Alba O.; Colas O.; Beck A.; Guillarme D.; Cianférani S. Hyphenation of Size Exclusion Chromatography to Native Ion Mobility Mass Spectrometry for the Analytical Characterization of Therapeutic Antibodies and Related Products. J. Chromatogr. B Analyt. Technol. Biomed. Life Sci. 2018, 1086, 176–183. 10.1016/j.jchromb.2018.04.010. 29684909

[ref34] Brinson R. G.; Ghasriani H.; Hodgson D. J.; Adams K. M.; McEwen I.; Freedberg D. I.; Chen K.; Keire D. A.; Aubin Y.; Marino J. P. Application of 2D-NMR with Room Temperature NMR Probes for the Assessment of the Higher Order Structure of Filgrastim. J. Pharm. Biomed. Anal. 2017, 141, 229–233. 10.1016/j.jpba.2017.03.063. 28454057 PMC6057790

[ref35] Ghasriani H.; Hodgson D. J.; Brinson R. G.; McEwen I.; Buhse L. F.; Kozlowski S.; Marino J. P.; Aubin Y.; Keire D. A. Precision and Robustness of 2D-NMR for Structure Assessment of Filgrastim Biosimilars. Nat. Biotechnol. 2016, 34, 139–141. 10.1038/nbt.3474. 26849514 PMC5218811

[ref36] Arbogast L. W.; Brinson R. G.; Formolo T.; Hoopes J. T.; Marino J. P. 2D 1HN, 15N Correlated NMR Methods at Natural Abundance for Obtaining Structural Maps and Statistical Comparability of Monoclonal Antibodies. Pharm. Res. 2016, 33, 462–475. 10.1007/s11095-015-1802-3. 26453189

[ref37] Arbogast L. W.; Delaglio F.; Tolman J. R.; Marino J. P. Selective Suppression of Excipient Signals in 2D 1H-13C Methyl Spectra of Biopharmaceutical Products. J. Biomol. NMR 2018, 72, 149–161. 10.1007/s10858-018-0214-1. 30483914

[ref38] Arbogast L. W.; Delaglio F.; Brinson R. G.; Marino J. P. Assessment of the Higher-Order Structure of Formulated Monoclonal Antibody Therapeutics by 2D Methyl Correlated NMR and Principal Component Analysis. Curr. Protoc. Protein Sci. 2020, 100, e105 10.1002/cpps.105. 32407007 PMC8288048

[ref39] Wu K.; Luo J.; Zeng Q.; Dong X.; Chen J.; Zhan C.; Chen Z.; Lin Y. Improvement in Signal-to-Noise Ratio of Liquid-State NMR Spectroscopy via a Deep Neural Network DN-Unet. Anal. Chem. 2021, 93, 1377–1382. 10.1021/acs.analchem.0c03087. 33377773

[ref40] Pastore A.; Salvadori S.; Temussi P. A. Peptides and Proteins in a Confined Environment: NMR Spectra at Natural Isotopic Abundance. J. Pept. Sci. 2007, 13, 342–347. 10.1002/psc.848. 17436341

[ref41] Sass H.-J.; Musco G.; Stahl S. J.; Wingfield P. T.; Grzesiek S. Solution NMR of Proteins within Polyacrylamide Gels: Diffusional Properties and Residual Alignment by Mechanical Stress or Embedding of Oriented Purple Membranes. J. Biomol. NMR 2000, 18, 303–309. 10.1023/A:1026703605147. 11200524

[ref42] Barrientos L. G.; Dolan C.; Gronenborn A. M. Characterization of Surfactant Liquid Crystal Phases Suitable for Molecular Alignment and Measurement of Dipolar Couplings. J. Biomol. NMR 2000, 16, 329–337. 10.1023/A:1008356618658. 10826884

[ref43] Tycko R.; Blanco F. J.; Ishii Y. Alignment of Biopolymers in Strained Gels: A New Way To Create Detectable Dipole–Dipole Couplings in High-Resolution Biomolecular NMR. J. Am. Chem. Soc. 2000, 122, 9340–9341. 10.1021/ja002133q.

[ref44] Lecoq L.; Fogeron M.-L.; Meier B. H.; Nassal M.; Böckmann A. Solid-State NMR for Studying the Structure and Dynamics of Viral Assemblies. Viruses 2020, 12 (), 10.3390/v12101069. PMC7599928 32987909

[ref45] Wiegand T.; Lacabanne D.; Torosyan A.; Boudet J.; Cadalbert R.; Allain F. H.-T.; Meier B. H.; Böckmann A. Sedimentation Yields Long-Term Stable Protein Samples as Shown by Solid-State NMR. Front. Mol. Biosci. 2020, 7, 17 10.3389/fmolb.2020.00017. 32154263 PMC7047159

[ref46] Hassan A.; Quinn C. M.; Struppe J.; Sergeyev I. V.; Zhang C.; Guo C.; Runge B.; Theint T.; Dao H. H.; Jaroniec C. P.; Berbon M.; Lends A.; Habenstein B.; Loquet A.; Kuemmerle R.; Perrone B.; Gronenborn A. M.; Polenova T. Sensitivity Boosts by the CPMAS Cryo Probe for Challenging Biological Assemblies. J. Magn. Reson. 2020, 311, 106680 10.1016/j.jmr.2019.106680. 31951864 PMC7060763

[ref47] Lu M.; Russell R. W.; Bryer A. J.; Quinn C. M.; Hou G.; Zhang H.; Schwieters C. D.; Perilla J. R.; Gronenborn A. M.; Polenova T. Atomic-Resolution Structure of HIV-1 Capsid Tubes by Magic-Angle Spinning NMR. Nat. Struct. Mol. Biol. 2020, 27, 863–869. 10.1038/s41594-020-0489-2. 32901160 PMC7490828

[ref48] Eddy M. T.; Yu T.-Y.; Wagner G.; Griffin R. G. Structural Characterization of the Human Membrane Protein VDAC2 in Lipid Bilayers by MAS NMR. J. Biomol. NMR 2019, 73, 451–460. 10.1007/s10858-019-00242-8. 31407201 PMC6819253

[ref49] Gupta R.; Zhang H.; Lu M.; Hou G.; Caporini M.; Rosay M.; Maas W.; Struppe J.; Ahn J.; Byeon I.-J. L.; Oschkinat H.; Jaudzems K.; Barbet-Massin E.; Emsley L.; Pintacuda G.; Lesage A.; Gronenborn A. M.; Polenova T. Dynamic Nuclear Polarization Magic-Angle Spinning Nuclear Magnetic Resonance Combined with Molecular Dynamics Simulations Permits Detection of Order and Disorder in Viral Assemblies. J. Phys. Chem. B 2019, 123, 5048–5058. 10.1021/acs.jpcb.9b02293. 31125232 PMC6767624

[ref50] le Paige U. B.; Xiang S.; Hendrix M. M. R. M.; Zhang Y.; Folkers G. E.; Weingarth M.; Bonvin A. M. J. J.; Kutateladze T. G.; Voets I. K.; Baldus M.; van Ingen H. Characterization of Nucleosome Sediments for Protein Interaction Studies by Solid-State NMR Spectroscopy. Magn. Reson. 2021, 2, 187–202. 10.5194/mr-2-187-2021. PMC9135053 35647606

[ref51] Mroue K. H.; MacKinnon N.; Xu J.; Zhu P.; McNerny E.; Kohn D. H.; Morris M. D.; Ramamoorthy A. High-Resolution Structural Insights into Bone: A Solid-State NMR Relaxation Study Utilizing Paramagnetic Doping. J. Phys. Chem. B 2012, 116, 11656–11661. 10.1021/jp307935g. 22953757 PMC3460063

[ref52] Azaïs T.; Von Euw S.; Ajili W.; Auzoux-Bordenave S.; Bertani P.; Gajan D.; Emsley L.; Nassif N.; Lesage A. Structural Description of Surfaces and Interfaces in Biominerals by DNP SENS. Solid State Nucl. Magn. Reson. 2019, 102, 2–11. 10.1016/j.ssnmr.2019.06.001. 31216494

[ref53] Cerofolini L.; Giuntini S.; Louka A.; Ravera E.; Fragai M.; Luchinat C. High-Resolution Solid-State NMR Characterization of Ligand Binding to a Protein Immobilized in a Silica Matrix. J. Phys. Chem. B 2017, 121, 8094–8101. 10.1021/acs.jpcb.7b05679. 28762736

[ref54] Louka A.; Matlahov I.; Giuntini S.; Cerofolini L.; Cavallo A.; Pillozzi S.; Ravera E.; Fragai M.; Arcangeli A.; Ramamoorthy A.; Goobes G.; Luchinat C. Engineering L-Asparaginase for Spontaneous Formation of Calcium Phosphate Bioinspired Microreactors. Phys. Chem. Chem. Phys. 2018, 20, 12719–12726. 10.1039/c8cp00419f. 29697113

[ref55] Ravera E.; Cerofolini L.; Martelli T.; Louka A.; Fragai M.; Luchinat C. (1) H-Detected Solid-State NMR of Proteins Entrapped in Bioinspired Silica: A New Tool for Biomaterials Characterization. Sci. Rep. 2016, 6, 27851 10.1038/srep27851. 27279168 PMC4899708

[ref56] Martelli T.; Ravera E.; Louka A.; Cerofolini L.; Hafner M.; Fragai M.; Becker C. F. W.; Luchinat C. Atomic-Level Quality Assessment of Enzymes Encapsulated in Bioinspired Silica. Chemistry 2016, 22, 425–432. 10.1002/chem.201503613. 26625942

[ref57] Fragai M.; Luchinat C.; Martelli T.; Ravera E.; Sagi I.; Solomonov I.; Udi Y. SSNMR of Biosilica-Entrapped Enzymes Permits an Easy Assessment of Preservation of Native Conformation in Atomic Detail. Chem. Commun. 2014, 50, 421–423. 10.1039/c3cc46896h. 24248259

[ref58] Ravera E.; Schubeis T.; Martelli T.; Fragai M.; Parigi G.; Luchinat C. NMR of Sedimented, Fibrillized, Silica-Entrapped and Microcrystalline (Metallo)Proteins. J. Magn. Reson. 2015, 253, 60–70. 10.1016/j.jmr.2014.12.019. 25797005

[ref59] Cerofolini L.; Giuntini S.; Carlon A.; Ravera E.; Calderone V.; Fragai M.; Parigi G.; Luchinat C. Characterization of PEGylated Asparaginase: New Opportunities from NMR Analysis of Large PEGylated Therapeutics. Chem. – Eur. J. 2019, 25, 1984–1991. 10.1002/chem.201804488. 30462348

[ref60] Cerofolini L.; Fragai M.; Ravera E.; Diebolder C. A.; Renault L.; Calderone V. Integrative Approaches in Structural Biology: A More Complete Picture from the Combination of Individual Techniques. Biomolecules 2019, 9, 370 10.3390/biom9080370. PMC6723403 31416261

[ref61] Giuntini S.; Balducci E.; Cerofolini L.; Ravera E.; Fragai M.; Berti F.; Luchinat C. Characterization of the Conjugation Pattern in Large Polysaccharide–Protein Conjugates by NMR Spectroscopy. Angew. Chem., Int. Ed. 2017, 56, 14997–15001. 10.1002/anie.201709274. PMC5813213 29024352

[ref62] Ravera E.; Ciambellotti S.; Cerofolini L.; Martelli T.; Kozyreva T.; Bernacchioni C.; Giuntini S.; Fragai M.; Turano P.; Luchinat C. Solid-State NMR of PEGylated Proteins. Angew. Chem. Int. Ed. Engl. 2016, 55, 2446–2449. 10.1002/anie.201510148. 26756539

[ref63] Giuntini S.; Cerofolini L.; Ravera E.; Fragai M.; Luchinat C. Atomic Structural Details of a Protein Grafted onto Gold Nanoparticles. Sci. Rep. 2017, 7, 17934 10.1038/s41598-017-18109-z. 29263419 PMC5738368

[ref64] Cerofolini L.; Giuntini S.; Ravera E.; Luchinat C.; Berti F.; Fragai M. Structural Characterization of a Protein Adsorbed on Aluminum Hydroxide Adjuvant in Vaccine Formulation. npj Vaccines 2019, 4, 20 10.1038/s41541-019-0115-7. 31149351 PMC6538755

[ref65] Viger-Gravel J.; Paruzzo F. M.; Cazaux C.; Jabbour R.; Leleu A.; Canini F.; Florian P.; Ronzon F.; Gajan D.; Lesage A. Atomic-Scale Description of Interfaces between Antigen and Aluminum-Based Adjuvants Used in Vaccines by Dynamic Nuclear Polarization (DNP) Enhanced NMR Spectroscopy. Chemistry 2020, 26, 8976–8982. 10.1002/chem.202001141. 32428253

[ref66] Jaudzems K.; Kirsteina A.; Schubeis T.; Casano G.; Ouari O.; Bogans J.; Kazaks A.; Tars K.; Lesage A.; Pintacuda G. Structural Analysis of an Antigen Chemically-Coupled on Virus-Like Particles in Vaccine Formulation. Angew. Chem. Int. Ed. Engl. 2021, 60, 12847 10.1002/anie.202013189. 33750007

[ref67] Wang H.; Wang L.; Li C.; Wuxiao Z.; Chen G.; Luo W.; Lu Y. Pegaspargase Combined with Concurrent Radiotherapy for Early-Stage Extranodal Natural Killer/T-Cell Lymphoma, Nasal Type: A Two-Center Phase II Study. Oncologist 2020, 25, e1725–e1731. 10.1634/theoncologist.2020-0144. 32627928 PMC7648361

[ref68] Herbert J.; Wilcox J. N.; Pham K.-T. C.; Fremeau R. T.; Zeviani M.; Dwork A.; Soprano D. R.; Makover A.; Goodman D. S.; Zimmerman E. A.; Roberts J. L.; Schon E. A. Transthyretin: A Choroid Plexus-Specific Transport Protein in Human Brain: The 1986 S. Weir Mitchell Award. Neurology 1986, 36, 900–900. 10.1212/WNL.36.7.900. 3714052

[ref69] Hamilton J. A.; Benson M. D. Transthyretin: A Review from a Structural Perspective. Cell. Mol. Life Sci. 2001, 58, 1491–1521. 10.1007/PL00000791. 11693529 PMC11337270

[ref70] Connors L. H.; Lim A.; Prokaeva T.; Roskens V. A.; Costello C. E. Tabulation of Human Transthyretin (TTR) Variants, 2003. Amyloid 2003, 10, 160–184. 10.3109/13506120308998998. 14640030

[ref71] Walker K. W.; Foltz I. N.; Wang T.; Salimi-Moosavi H.; Bailis J. M.; Lee F.; An P.; Smith S.; Bruno R.; Wang Z. The Serum Protein Transthyretin as a Platform for Dimerization and Tetramerization of Antibodies and Fab Fragments to Enable Target Clustering. J. Biol. Chem. 2020, 295, 10446–10455. 10.1074/jbc.RA120.013135. 32518163 PMC7383373

[ref72] Fragai M.; Luchinat C.; Parigi G.; Ravera E. Practical Considerations over Spectral Quality in Solid State NMR Spectroscopy of Soluble Proteins. J. Biomol. NMR 2013, 57, 155–166. 10.1007/s10858-013-9776-0. 23990200

[ref73] Sarker B.; Papageorgiou D. G.; Silva R.; Zehnder T.; Gul-E-Noor F.; Bertmer M.; Kaschta J.; Chrissafis K.; Detsch R.; Boccaccini A. R. Fabrication of Alginate-Gelatin Crosslinked Hydrogel Microcapsules and Evaluation of the Microstructure and Physico-Chemical Properties. J. Mater. Chem. B 2014, 2, 1470–1482. 10.1039/c3tb21509a. 32261366

[ref74] Wang Q.-Q.; Liu Y.; Zhang C.-J.; Zhang C.; Zhu P. Alginate/Gelatin Blended Hydrogel Fibers Cross-Linked by Ca2+ and Oxidized Starch: Preparation and Properties. Mater. Sci. Eng. C Mater. Biol. Appl. 2019, 99, 1469–1476. 10.1016/j.msec.2019.02.091. 30889681

[ref75] Schuetz A.; Wasmer C.; Habenstein B.; Verel R.; Greenwald J.; Riek R.; Böckmann A.; Meier B. H. Protocols for the Sequential Solid-State NMR Spectroscopic Assignment of a Uniformly Labeled 25 KDa Protein: HET-s (1-227). ChemBioChem 2010, 11, 1543–1551. 10.1002/cbic.201000124. 20572250

[ref76] Lu X.; Guo C.; Hou G.; Polenova T. Combined Zero-Quantum and Spin-Diffusion Mixing for Efficient Homonuclear Correlation Spectroscopy under Fast MAS: Broadband Recoupling and Detection of Long-Range Correlations. J. Biomol. NMR 2015, 61, 7–20. 10.1007/s10858-014-9875-6. 25420598 PMC4485404

[ref77] Keller R. The Computer Aided Resonance Assignment Tutorial (CARA); The CARA/Lua Programmers Manual. DATONAL AG.; CANTINA Verlag: Goldau. Switzerland, 2004.

[ref78] Leach J. B.; Schmidt C. E. Characterization of Protein Release from Photocrosslinkable Hyaluronic Acid-Polyethylene Glycol Hydrogel Tissue Engineering Scaffolds. Biomaterials 2005, 26, 125–135. 10.1016/j.biomaterials.2004.02.018. 15207459

[ref79] Jia J.; Richards D. J.; Pollard S.; Tan Y.; Rodriguez J.; Visconti R. P.; Trusk T. C.; Yost M. J.; Yao H.; Markwald R. R.; Mei Y. Engineering Alginate as Bioink for Bioprinting. Acta Biomater. 2014, 10, 4323–4331. 10.1016/j.actbio.2014.06.034. 24998183 PMC4350909

[ref80] Ouyang L.; Yao R.; Zhao Y.; Sun W. Effect of Bioink Properties on Printability and Cell Viability for 3D Bioplotting of Embryonic Stem Cells. Biofabrication 2016, 8, 035020 10.1088/1758-5090/8/3/035020. 27634915

[ref81] Gao T.; Gillispie G. J.; Copus J. S.; Pr A. K.; Seol Y.-J.; Atala A.; Yoo J. J.; Lee S. J. Optimization of Gelatin-Alginate Composite Bioink Printability Using Rheological Parameters: A Systematic Approach. Biofabrication 2018, 10, 034106 10.1088/1758-5090/aacdc7. 29923501 PMC6040670

[ref82] Liao Y.-H.; Jones S. A.; Forbes B.; Martin G. P.; Brown M. B. Hyaluronan: Pharmaceutical Characterization and Drug Delivery. Drug Delivery 2005, 12, 327–342. 10.1080/10717540590952555. 16253949

