# Supplementary material for: Evaluation of the Higher Order Structure of Biotherapeutics Embedded in Hydrogels for Bioprinting and Drug Release
Source: Anal Chem. 2021 Aug 2;93(32):11208–14. doi: 10.1021/acs.analchem.1c01850 (PMC8382223; doi:10.1021/acs.analchem.1c01850)
Supplement: Supplementary file 1 — ac1c01850_si_001.pdf [file ac1c01850_si_001.pdf]

Supporting Information

Evaluation of the Higher Order Structure of biotherapeutics embedded in hydrogels for bioprinting and drug release

Domenico Rizzo[1,2], Linda Cerofolini[1], Anna Pérez-Ràfols[2,3], Stefano Giuntini[1,2], Fabio Baroni[4], Enrico Ravera[1,2], Claudio Luchinat[1,2], Marco Fragai[*1,2]

1. Magnetic Resonance Center (CERM), University of Florence, and Consorzio Interuniversitario Risonanze Magnetiche di Metalloproteine (CIRMMP) Via L. Sacconi 6, 50019 Sesto Fiorentino, Italy
2. Department of Chemistry "Ugo Schiff", University of Florence, Via della Lastruccia 3, 50019, Sesto Fiorentino, Italy
3. Giotto Biotech, S.R.L, Via Madonna del piano 6, 50019, Sesto Fiorentino (FI), Italy
4. Analytical Development Biotech Department, Merck Serono S.p.a, Guidonia, RM, Italy; an affiliate of Merck KGaA

*Corresponding author; Phone: +39 055 4574261; E-mail: fragai@cerm.unifi.it

Table of Contents

Expression and purification of uniformly isotopically enriched ANSII [U-^{13}C-^{15}N] S2

Expression and purification of uniformly isotopically enriched TTR [U-^{13}C-^{15}N] S2

Figure S1 S4

Figure S2 S5

Figure S3 S6

Figure S4 S7

Expression and purification of uniformly isotopically enriched ANSII [U-^{13}C-^{15}N]. Escherichia coli C41(DE3) cells were transformed with pET-21a(+) plasmid encoding ANSII gene. The cells were cultured in ^{13}C-, ^{15}N-enriched minimal medium (M9) containing 0.1 mg/mL of ampicillin, and grown at 310 K until OD_{600} reached 0.6–0.8. Then, the cells were induced with 1 mM isopropyl β-D-1-thiogalactopyranoside and further grown at 310 K overnight. Finally, they were harvested by centrifugation at 6500 rpm (JA-10 Beckman Coulter) for 15 min at 277 K. The pellet obtained from 1 liter of culture was suspended in 60 mL of 10 mM Tris-HCl buffer, at pH 8.0, with 15 mM EDTA, 20% sucrose and incubated at 277 K for 20 min, under magnetic stirring. The suspension was centrifuged at 10,000 rpm (F15-6x100y Thermo Scientific) for 30 min, and the supernatant discarded. The recovered pellet was re-suspended in H_2O milli-Q and newly incubated with the Tris-HCl buffer solution at 277 K for 20 min under magnetic stirring. Again, the suspension was centrifuged at 10,000 rpm (F15-6x100y Thermo Scientific) for 30 min. The pellet was discarded, whereas the supernatant was treated with ammonium sulfate. Still under magnetic stirring, aliquots of solid ammonium sulfate were added up to 50% saturation. Then, the precipitate was removed by centrifugation, and ammonium sulfate added again up to 90% saturation to trigger the precipitation of ANSII, which was recovered by centrifugation. The precipitated ANSII was re-dissolved in a minimal amount of 20 mM Tris-HCl buffer at pH 8.6 and dialyzed extensively against the same buffer. ANSII was purified by anionic-exchange chromatography using a HiPrep Q FF 16/10 column (GE Healthcare Life Science). The protein was eluted in 20 mM Tris-HCl buffer at pH 8.6 with a linear 0–1 M NaCl gradient. Fractions containing pure ANSII were identified by Coomassie staining SDS-PAGE gels, then joined and dialyzed extensively against 0.5 mM Tris-HCl buffer at pH 7.5. Aliquots of 0.5 mL, each containing 1 mg of protein, were freeze-dried to be used for SSNMR analysis.

Expression and purification of uniformly isotopically enriched TTR [U-^{13}C-^{15}N]. Escherichia coli BL21(DE3) RIPL pLysS cells were transformed with pET-28a(+) plasmid encoding TTR gene. The cells were cultured in ^{13}C-, ^{15}N-enriched minimal medium (M9) containing 0.1 mg/mL of kanamycin, grown at 310 K, until OD_{600} reached 0.6–0.8 and then induced with 1 mM isopropyl β-D-1-thiogalactopyranoside. The cells were further grown at 310 K overnight and then harvested by centrifugation at 6500 rpm (JA-10 Beckman Coulter) for 15 min at 277 K. The pellet was suspended in 20 mM Tris-HCl at pH 8.5 (60 mL per liter of culture) and sonicated at 277 K for 40 min. The suspension was centrifuged at 40,000 rpm (F15-6x100y Thermo Scientific) for 40 min and the pellet discarded. The protein was purified by anionic-exchange chromatography using a HiPrep Q FF 16/10 column (GE Healthcare Life Science). The protein was eluted in 20 mM Tris-HCl buffer at pH 8.6 with a linear 0–1 M NaCl gradient. Fractions containing pure TTR were identified by Coomassie

staining SDS-PAGE gels, then joined and purified by Size Exclusion Chromatography using HiLoad Superdex 26/60 75pg in 50 mM phosphate buffer at pH 7.5; then dialyzed extensively against 2 mM Tris-HCl buffer at pH 7.5. Aliquots of 1 mL, each containing 6 mg of protein, were freeze-dried to be used for SSNMR analysis.

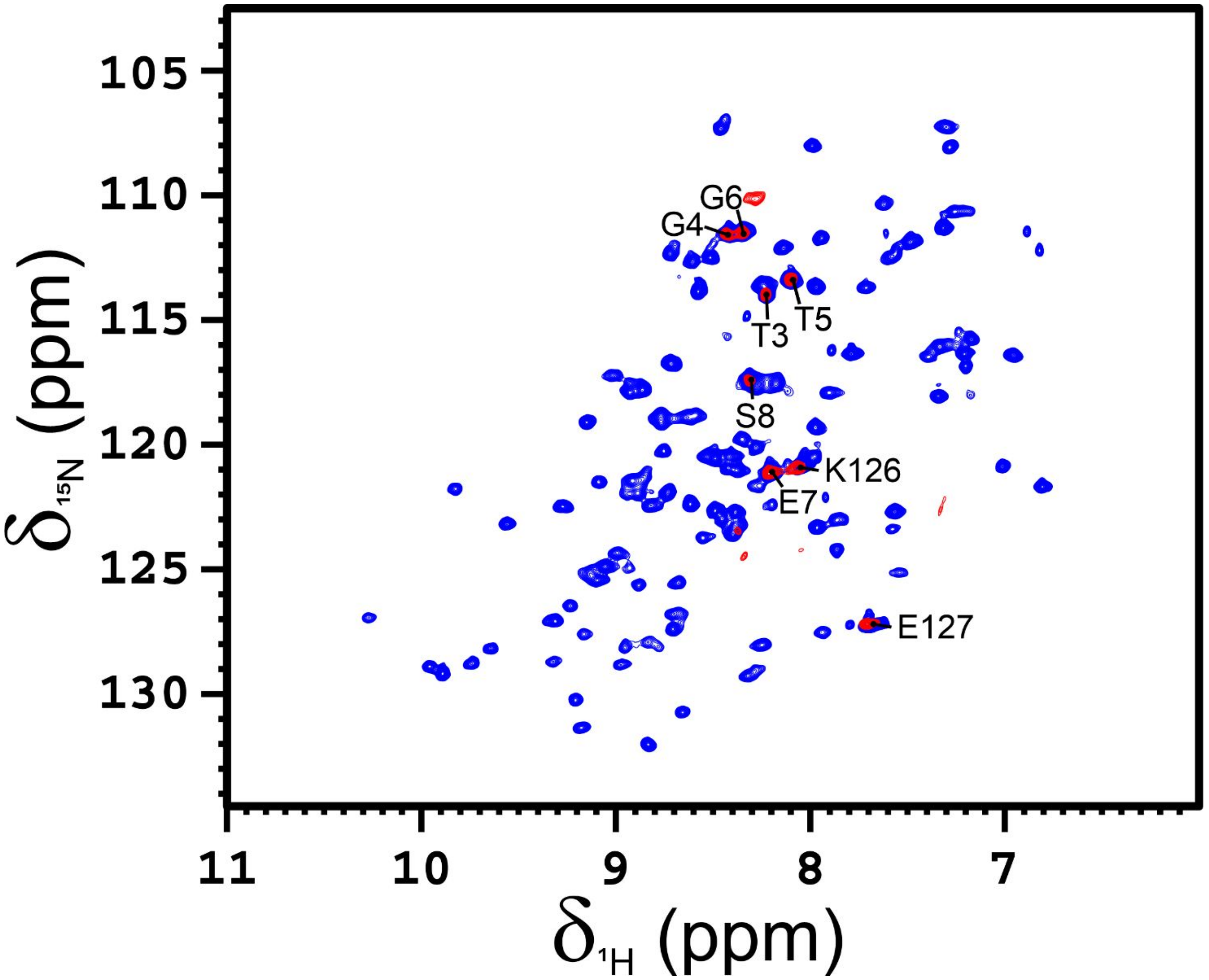

Figure S1. 2D ^{1}H-^{15}N TROSY-HSQC spectrum of TTR (blue) collected on a solution of the protein at the concentration of 100 μM superimposed with the same spectrum collected on TTR (100 μM) encapsulated in alginate/gelatine hydrogel (red). The spectra were recorded on a 950 MHz spectrometer at 310 K.

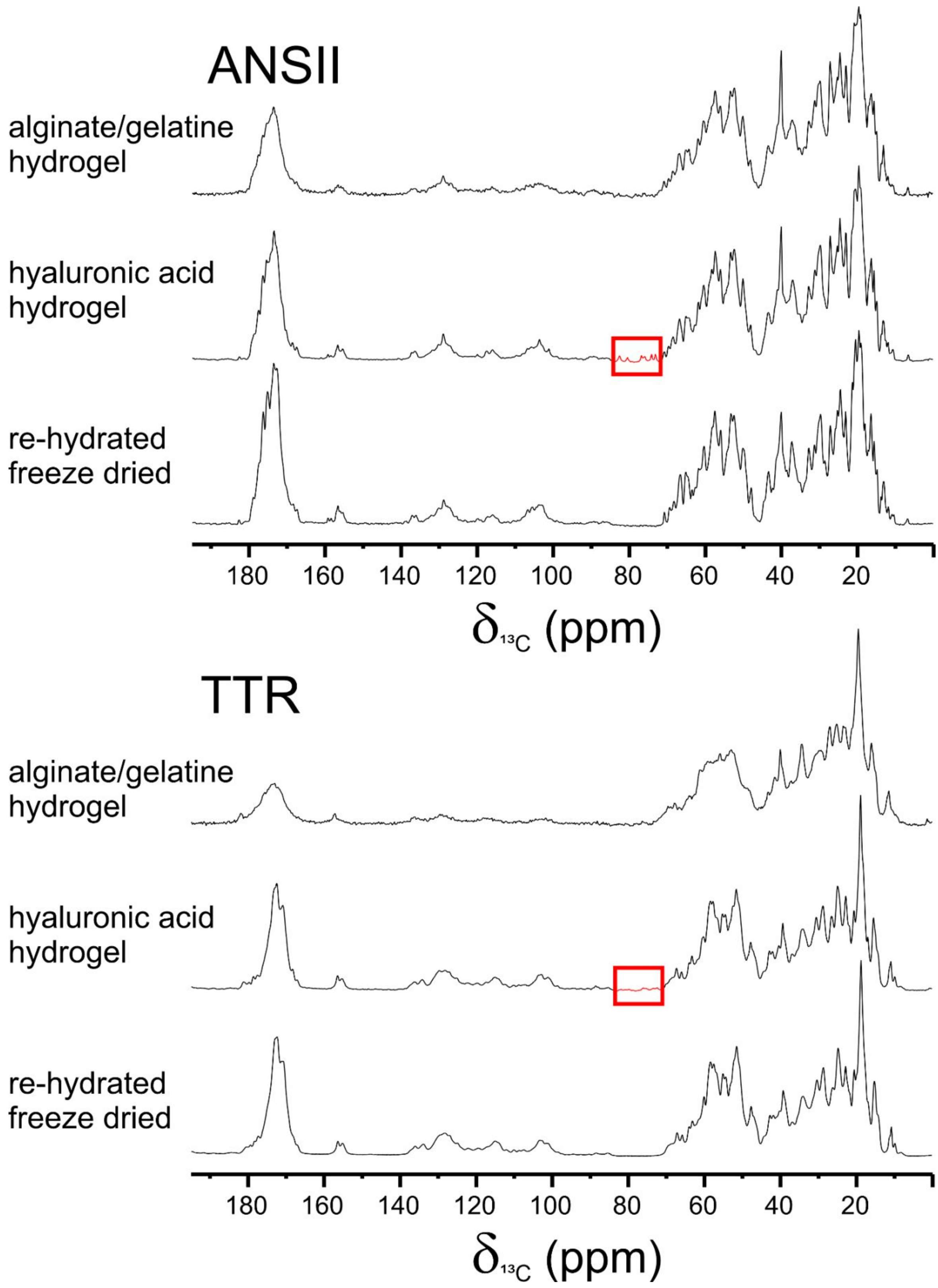

Figure S2. 1D {^{1}H}^{13}C cross-polarization spectra acquired on the re-hydrated freeze-dried proteins, on the proteins embedded in the hyaluronic acid hydrogels, and in the alginate/gelatine hydrogels (ANSII, top, and TTR, bottom). The signals of the hyaluronic acid are highlighted by a red box. The spectra were acquired at ~290 K, MAS 14 kHz and 800 MHz (number of scans: 512, recycle delay: 3 sec).

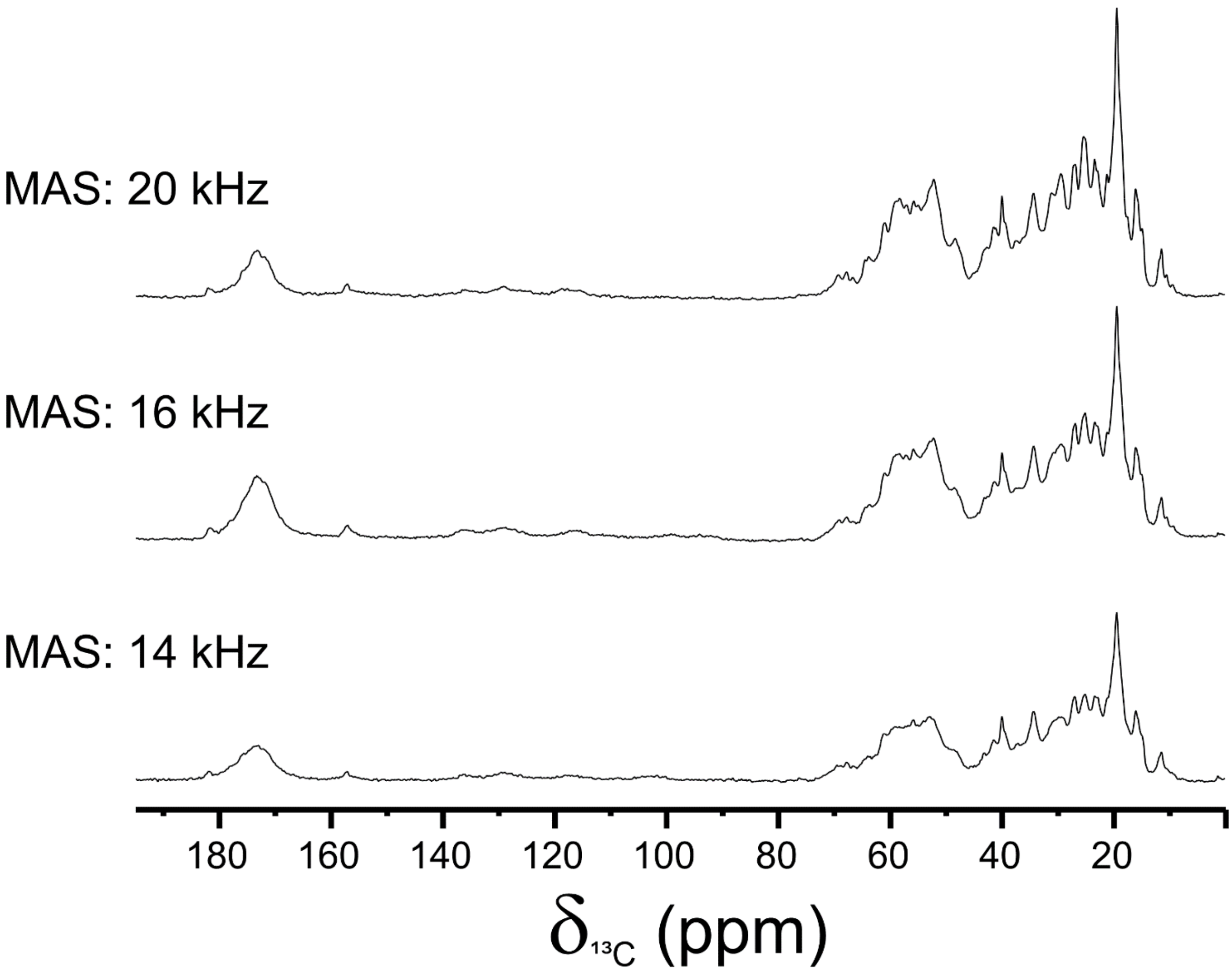

Figure S3. 1D {^{1}H}^{13}C cross-polarization spectra acquired on the TTR-AG at the different MAS speeds indicated in the figure. The spectra were acquired at ~290 K and 800 MHz (number of scans: 512, recycle delay: 3 sec).

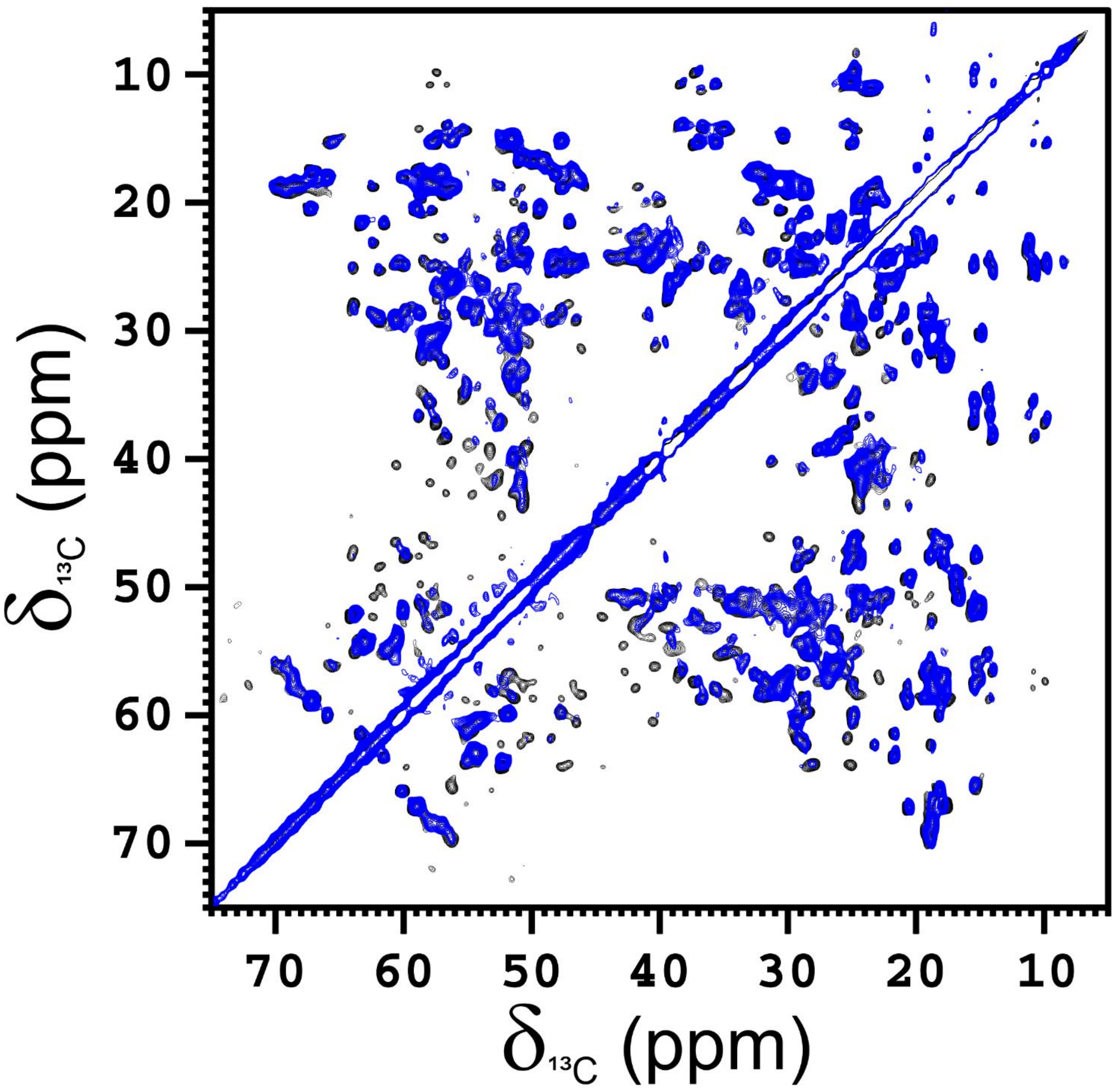

Figure S4. Aliphatic region of the 2D ^{13}C-^{13}C CORDxy4 recorded on TTR-AG hydrogel (blue) superimposed with the CORDxy4 recorded on the re-hydrated freeze-dried TTR (black). The spectra were acquired at ~290 K, MAS 20 kHz and 800 MHz.
